# Pan-cancer analysis for the prognostic and immunological role of CD47: interact with TNFRSF9 inducing CD8 + T cell exhaustion

**DOI:** 10.1007/s12672-024-00951-z

**Published:** 2024-05-08

**Authors:** Hongxin Liang, Yong Zheng, Zekai Huang, Jinchi Dai, Lintong Yao, Daipeng Xie, Duo Chen, Hongrui Qiu, Huili Wang, Hao Li, Jinhang Leng, Ziming Tang, Dongkun Zhang, Haiyu Zhou

**Affiliations:** 1 Guangdong Provincial People’s Hospital, Guangdong Cardiovascular Institute, Guangdong Academy of Medical Sciences, Guangzhou, 510100 China; 2 Department of Anesthesiology, Guangdong Provincial People’s Hospital (Guangdong Academy of Medical Sciences), Southern Medical University, Guangzhou, 510080 China; 3 The First School of Clinical Medicine, Guangdong Medical University, Zhanjiang, 524023 China; 4 Department of Thoracic Surgery, Guangdong Provincial People’s Hospital (Guangdong Academy of Medical Sciences), Southern Medical University, Guangzhou, 510080 China; 5 Southern Medical University, Guangzhou, 510515 China; 6 Department of Respiratory and Critical Care Medicine, Beijing Institute of Respiratory Medicine and Beijing Chao-Yang Hospital, Capital Medical University, Beijing, 100020 China

**Keywords:** CD47, CD8 + T cells, T-cell exhausted, Pan-cancer

## Abstract

**Purpose:**

The research endeavors to explore the implications of CD47 in cancer immunotherapy effectiveness. Specifically, there is a gap in comprehending the influence of CD47 on the tumor immune microenvironment, particularly in relation to CD8 + T cells. Our study aims to elucidate the prognostic and immunological relevance of CD47 to enhance insights into its prospective utilities in immunotherapeutic interventions.

**Methods:**

Differential gene expression analysis, prognosis assessment, immunological infiltration evaluation, pathway enrichment analysis, and correlation investigation were performed utilizing a combination of R packages, computational algorithms, diverse datasets, and patient cohorts. Validation of the concept was achieved through the utilization of single-cell sequencing technology.

**Results:**

CD47 demonstrated ubiquitous expression across various cancer types and was notably associated with unfavorable prognostic outcomes in pan-cancer assessments. Immunological investigations unveiled a robust correlation between CD47 expression and T-cell infiltration rather than T-cell exclusion across multiple cancer types. Specifically, the CD47-high group exhibited a poorer prognosis for the cytotoxic CD8 + T cell Top group compared to the CD47-low group, suggesting a potential impairment of CD8 + T cell functionality by CD47. The exploration of mechanism identified enrichment of CD47-associated differentially expressed genes in the CD8 + T cell exhausted pathway in multiple cancer contexts. Further analyses focusing on the CD8 TCR Downstream Pathway and gene correlation patterns underscored the significant involvement of TNFRSF9 in mediating these effects.

**Conclusion:**

A robust association exists between CD47 and the exhaustion of CD8 + T cells, potentially enabling immune evasion by cancer cells and thereby contributing to adverse prognostic outcomes. Consequently, genes such as CD47 and those linked to T-cell exhaustion, notably TNFRSF9, present as promising dual antigenic targets, providing critical insights into the field of immunotherapy.

**Supplementary Information:**

The online version contains supplementary material available at 10.1007/s12672-024-00951-z.

## Introduction

The advent of immunotherapies has brought about significant advancements in the survival rates of cancer patients. The upregulation of various immune checkpoints serves as a key mechanism facilitating tumor immune evasion and represents a primary barrier that hampers the effectiveness of immune-based treatments. CD47, a pivotal checkpoint of innate immunity, emerges as a focal point of investigation. Delving into the intricate interplay between CD47 and the tumor immune microenvironment (TIME) to delineate a comprehensive understanding of tumor immune evasion pathways holds promise for the development of tailored therapeutic interventions and the identification of novel immune targets. These endeavors are poised to address challenges associated with immune therapy resistance, ultimately enhancing the survival outcomes of individuals afflicted with malignancies.

CD47 is a transmembrane glycoprotein belonging to the immunoglobulin superfamily, known to interact with signal regulatory protein α(SIRPα). The CD47/SIRPα axis functions to impede myosin accumulation, thereby initiating the "Do not eat me" signal to evade phagocytosis by macrophages [1, 2] and suppress innate immunity [3–5]. Overexpression of CD47 in various tumor cell types has been identified as a mechanism for evading innate immunity[4], correlating with diminished survival outcomes and reduced responsiveness to conventional therapies[6–8]. Targeting the CD47/SIRPα axis has been a focal point of investigation, with therapeutic strategies including monoclonal antibodies (McAb), bispecific antibodies, fusion proteins, combination chemotherapies, and immunotherapies, such as CD47 McAb and fusion proteins incorporating SIRPα(e.g., TTI-621/2). These approaches have demonstrated notable clinical efficacy and are currently under evaluation in phase II or III clinical trials[9–11].

Significantly, the CD47/SIRPα axis exerts influence on adaptive immunity as well. Studies have revealed that inhibition of the CD47/SIRPα axis can directly enhance T-cell responses or act through modulation of myeloid cells [12, 13]. Notably, a subset of CD8 + T cells has been identified to express SIRPα[14], and disrupting the CD47/SIRPα interaction can overcome resistance to CD8-mediated immunotherapy [15, 16]. Additionally, CD47 agonists have demonstrated the ability to promote antigen presentation and facilitate cross-priming of T-lymphocytes [17, 18]. Preclinical mouse models have underscored the role of CD47 monoclonal antibodies in T-cell cross-priming, with a lack of therapeutic response observed in T-cell-deficient mice but rescued in wild-type counterparts [19]. Combining radiotherapy with CD47 blockade has been shown to enhance antitumor immunity by directly impacting CD8 + T cells [20]. The intricate interplay between the tumor microenvironment (TME), tumor immune evasion, cancer prognosis, and therapeutic responses has been elucidated [21, 22], highlighting the significance of targeting the CD47/SIRPα axis to impede phagocytosis and counteract innate immunity checkpoints associated with tumor immune evasion. Nonetheless, a comprehensive understanding of the implications of adaptive immunity in regulating the CD47 checkpoint and its impact on antitumor T-cell immunity remains an imperative area for further exploration.

A promising target of interest is the tumor necrosis factor (TNF) receptor superfamily member TNFRSF9 (CD137, TNFRSF9). TNFRSF9 expression is specifically induced through the interaction between the T cell receptor (TCR) and the major histocompatibility complex (MHC) [23]. Identified as a characteristic marker of tumor-reactive T cell subsets within the tumor microenvironment (TME), TNFRSF9 is notably absent on static T cells present in peripheral blood [24, 25]. DSP107, a fusion protein, exhibits dual immune regulatory capabilities by binding to both targets, thereby stimulating innate and adaptive immune responses and showcasing potent antitumor effects. Through the natural trimerization of DSP107 via the 4-1BBL trimerization domain, binding to CD47 on cancer cells disrupts the CD47-SIRPα interaction. This interaction also facilitates the immobilization of DSP107 on the cancer cell surface, enabling the delivery of the 4-1BBL-4-1BB costimulatory signal to T cells localized within the tumor microenvironment. The dual immunomodulatory mechanism of DSP107 is strategically formulated to activate both innate and adaptive immune responses at the tumor site, ultimately enhancing antitumor immunity [26].

This study conducted a comprehensive analysis of the prognostic and immunological implications of CD47 in pan-cancer, with a specific focus on its association with CD8 + T cell exhaustion. The investigation aimed to elucidate the potential impact of CD47 interaction with TNFRSF9 within the tumor immune microenvironment (TIME) on CD8 + T cell functionality and its consequent effect on the prognosis of patients with malignant tumors. Leveraging a range of bioinformatics tools, the analysis encompassed differential expression assessments, prognosis evaluations, immune cell infiltration patterns, and pathway enrichment analyses across various cancer types. Furthermore, the co-expression relationship between CD47 and the pivotal gene TNFRSF9 was validated utilizing single-cell sequencing data. The research design and technical approach are well-structured and reasonable.

## Methods

### Analysis tools and data collection

XENA-TCGA GTEx

TCGA (https://portal.gdc.cancer.gov/) and GTEx handling were consolidated by the Toil process in UCSC XENA (https://xenabrowser.net/datapages/). Data (V8.0) conversion: Transcripts per million reads format RNAseq data in TPM format and log2 conversion for analysis and comparison. GTEx, The Genotype-Tissue Expression (https://www.gtexportal.org/home/). After log2 transformation, RNAseq data in TPM (transcripts per million reads) format was examined and contrasted. [27]

UALCAN

A comprehensive OMICS cancer data analysis web portal is located in Ualcan (http://ualcan.path.uab.edu/). The expression level of CD47 was normalized as transcript per million reads. P < 0.05 was considered statistically significant [28].

TIMER2.0

TIMER2.0, Tumor IMmune Estimation Resource (https://cistrome.shinyapps.io/timer/) is a database for comprehensive analysis of tumor-infiltrating immune cells [29]. The TIMER database consists of 10897 samples from 32 TCGA cancer types to evaluate immune infiltrate abundance.

TIDE

TIDE [30] (http://tide.dfci.harvard.edu/) stands for Tumor Immune Dysfunction and Exclusion. It is a computational framework developed to evaluate the potential of tumor immune escape from the gene expression profiles of cancer samples. The TIDE score computed for each tumor sample can be a surrogate biomarker to predict response to immune checkpoint blockade, including anti-PD1 and anti-CTLA4 for melanoma and NSCLC. The highly scored genes in TIDE signatures also present potential regulators of tumor immune escape and resistance to cancer immunotherapies [31].

STRING

STRING database [32]: (string-db.org) is a protein interaction network database based on public database and literature information. It gathers several public databases, including UniProt, KEGG, NCBI, and Gene Ontology, to integrate these data and generate a comprehensive protein interaction network database.

TISCH

TISCH collected data from Gene Expression Omnibus (GEO) [33] and Array Express [34] to formulate its scRNA-seq atlas [35], including 79 databases and 2045746 cells from tumor patients and healthy donors. Data sets were processed uniformly to allow for clarification of the TME components at both the single-cell and annotated cluster levels.

HPA[36] (https://www.proteinatlas.org).

The HPA database (Human Protein Atlas) is based on proteomics, transcriptomics, and systems biology data to map tissues, cells, and organs. It includes not only tumor tissue, but also normal tissue protein expression, and can also check the survival curve of tumor patients.

STATISTIC

P value less than 0.05 was statistically significant. Significance markers: NS, P ≥ 0.05; *, p < 0.05; * *, p < 0.01; * * *, p < 0.001.

### Analysis of differential CD47 expression in average, tumor stages, and protein levels

Differential CD47 expression levels between tumors and normal tissues adjacent to TCGA cancer types were analyzed using R (version 3.6.3) and R packages (mainly GGGlot2 [version 3.3.3]) from the XENA-TCGA GTEx resource. Furthermore, Protein levels between tumors and adjacent normal tissues were also investigated using the UALCAN interactive web resource. Survival curves were presented using predictive analysis. SurvMiner [version 0.4.9] and Survival package [version 3.2–10] were used (grouped by p-best). The type of prognosis was OS (Overall Survival), DSS (Disease-Specific Survival), and prognostic data were also obtained from a Cell article [37], and finally verified by immunohistochemistry of HPA database.

### Analysis of tumor immune and immunosuppressive cell infiltration and comparative biomarker analysis

Using the TIMER2 server, we analyzed the correlation between tumor infiltration and CD47 expression, with four immunosuppressive cell types promoting T-cell rejection, MDSCs, CAF, M2-TAM, and Treg across 39 TCGA cancers. The Spearman partial rho value and p < 0.05 were used for correlation analysis. The results of the study used the algorithm with the best positive results. In addition, we used the GSVA R package [version 1.34.0] [38] to explore correlations between the expression of CD47 and the infiltration of 23 types of immune cells [39] in TCGA cancers. Then, the overall predictive power of CD47 was compared with standardized biomarkers of tumor immune response in terms of treatment response outcome and OS, and the correlation analysis of CD47 with other immune checkpoints, MHC class molecules and other immune-related molecules was explored.

### Analysis of CD47 on cytotoxic CD8 + T cell infiltration influenced tumor prognosis

We used the GSVA R package [version 1.34.0] [38, 39] to explore the difference in Cytotoxic T-cell infiltration in the different CD47 expression situations in TCGA pan-cancer. The median method was used to divide patients into CD47- high group and CD47-low group. We also use the TIDE algorithm to assess the effects of CD47 on Cytotoxic CD8 + T cell [31].

### Analysis of pathway

We identify DEG between high and low-expression CD47 clusters using the DESeq2 R package [1.26.0 version] [40]. Division of patients into high and low CD47 groups by median method. These different genes were enriched in the pathway via the R package cluster profile [41, 42] [3.14.3 version] (for SEEA analysis) [42]. [version 3.14.3] (for GSEA analysis) [42]. Reference gene set: H.all.v7.2.symbols.GMT [Hallmarks]. The species is Homo sapiens. Gene set databases came from MSigDB Collections [43]. It included the BIOCARTA subset of CP (browse 292 gene sets); KEGG subset of CP (browse 186 gene sets); PID subset of CP (browse 196 gene sets); REACTOME subset of CP (browse 1615 gene sets); WikiPathways subset of CP (browse 664 gene sets). Significance: It is generally considered that the conditions of False Discovery Rate (FDR) < 0.25 and p.adjust < 0.05. Visualization: GGploT2 [version 3.3.3]. Furthermore, CBNplot [44] was used to investigate molecular regulatory connections. in PID_CD8_TCR_DOWNSTREAM_PATHWAY, exhibiting a Bayesian network inference approach. STRING database was used to detect the PPI (Protein–Protein Interaction Networks), showing the interactions of CD47 protein and core protein of that pathway in biological systems.

### Analysis of co-expression.

We used Software: R (version 3.6.3) to analyze the correlation of CD47 and T-cell exhaustion in TCGA pan-cancer. The data sets were level 3 HTSeq—FPKM RNAseq data format. Visualization of the results is provided by the R package: GGploT2 [version 3.3.3]. Besides, we used TISCH to determine whether CD47 was mainly expressed primarily on CD8 + Tex and whether CD47 has a relationship with the CD8 + Tex gene expressed primarily on CD8 + Tex. The results were presented in the stacked value bar chart (bars of superimposed proportions) to see the expression levels of CD47 and TNFRSF9 in different cohorts through software R (version 3.6.3) and GGPLOT2 [version 3.3.3] (visualization).

## Results

### Study flowchart

**Fig. 1:**
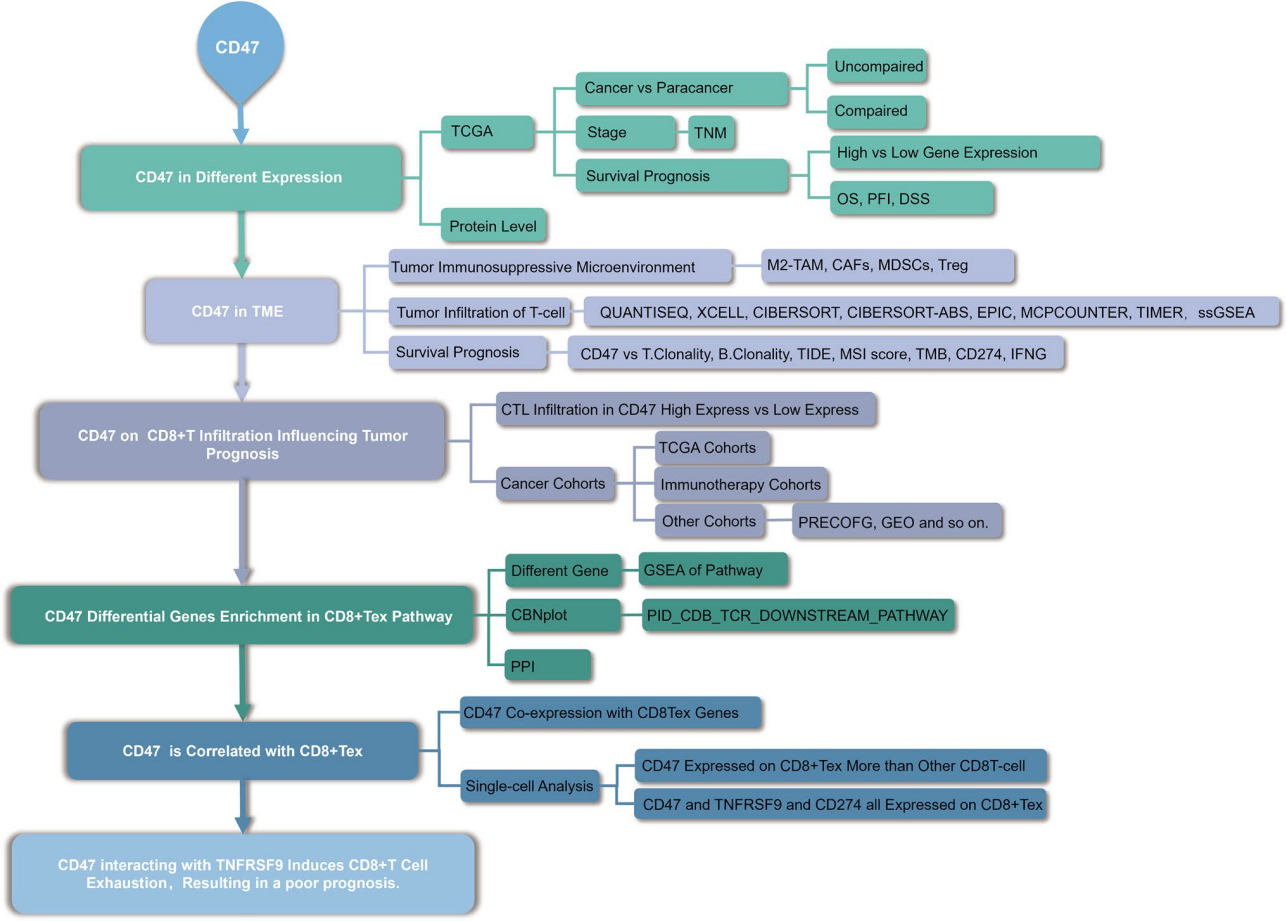
The workflow of the study. TCGA, The Cancer Genome Atlas; TME, Tumor microenvironment; TNM, Tumor Node Metastasis; TAMs, tumor-associated macrophages; CAFs, cancer-associated fibroblasts; aDC (activated DC); B cells; CD8 T cells; CTL(Cytotoxic T cells); DC; Eosinophils; iDC (immature DC); Macrophages; Mast cells; Neutrophils; NK CD56bright cells; NK CD56dim cells; NK cells; pDC (Plasmacytoid DC); T cells; T helper cells; Tcm (T central memory); Tem (T effector memory); Tfh (T follicular helper); Tgd (T gamma delta); Th1 cells; Th17 cells; Th2 cells; Treg, regulatory T cells; CAFs, cancer-associated fibroblasts; MDSCs, myeloid-derived suppressor cells; M2-TAMs; M2 subtype of tumor-associated macrophages. MSI, Microsatellite instability; TMB, Tumor mutational burden; CD274, Cluster of differentiation 274; IFNG, interferon-γ; 4-1BB(TNFRSF9), TNF receptor superfamily member 9; CD8 + Tex, exhausted CD8 T lymphocyte cell

Figure 1 illustrates the schematic diagram of the present study. The study encompasses clinic prognosis and gene data analyses.

### Abnormal expression of CD47 in pan-cancer patients is associated with tumor stages and poor prognosis

**Fig. 2:**
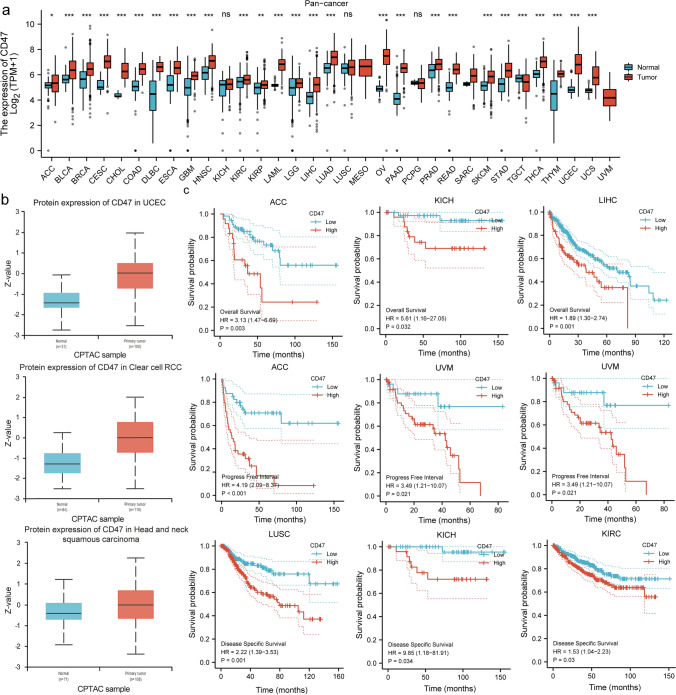
CD47 is aberrantly overexpressed and is associated with poor cancer prognoses. (**A**) Boxplots showing differential CD47 expression levels (log2FPKM + 1)/ (log2TPM + 1) between tumors in the XENA-TCGA_GTEx database. Box plots showing differential CD47 expression levels (log2FPKM + 1)/ (log2TPM + 1) between tumor and adjacent normal tissues (Paired Patient) across the TCGA database. CD47 is expressed differently in multiple cancers. **B** Boxplots illustrating the varying levels of CD47 expression (protein) among tumors in the CPTAC database. **C** Kaplan–Meier curves of cumulative survival differences between TCGA cancer cohorts with high and those with low expression levels of CD47. The presentation showcases TCGA cancers that exhibit statistically significant variations among the cohorts. UCSC XENA (https://xenabrowser.net/datapages/) by the Toil process unified TCGA RNAseq TPM format data processing. (GTEx)The Genotype-Tissue Expression; Significance representation: ns, p ≥ 0.05; *, p < 0.05; **, p < 0.01; ***, p < 0.001

We looked into CD47's carcinogenic potential using the XENA-TCGA GTEx pan-cancer database. When comparing nearly all cancer types to normal tissue, we discovered that CD47 gene expression was higher in the former. (ACC, BRCA, BLCA, CHOL, COAD, DLBC, ESCA, GBM, HNSC, KIRC, KIRP, LAML, LGG, LIHC, LUAD, OV, PAAD, PRAD, READ, SARC, SKCM, STAD, THCA, THYM, UCEC, UCS) (Fig. 2a). Additionally, we delved deeper into the CD47 expression of paired samples within the XENA-TCGA database, yielding identical outcomes to the XENA-TCGA GTEx pan-cancer database across various cancer types including BRCA, CHOL, COAD, ESCA, HNSC, KIRC, LIHC, PAAD, STAD, THCA, and UCEC. (Supplementary Fig. 1a).

Significantly, we noted an increase in CD47 protein expression in HNSC, PAAD, UCEC, RCC, and OV compared to the normal levels, as indicated by the UALCAN database (Fig. S2b, Supplementary Fig. 1b). Moreover, in cancer, the expression of CD47 was elevated in advanced tumor stages. For example, patients with M1 stage lung squamous cell carcinoma malignancy expressed more CD47 than patients with M0 stage. The same trend's outcomes were observed in THCA, UCEC, PRAD, KIRC, KIRP, and LIHC. (Supplementary Fig. 1c). Subsequently, we discovered a correlation between excessive CD47 expression and reduced overall survival in ACC, BRCA, LIHC, and KICH; decreased PFI in ACC, LUSC, UVM, and decreased DSS in ACC, LUSC, LGG, and KICH. All of these findings point to CD47 could be an early biomarker for cancer detection, staging, and monitoring. (Fig. 2c, Supplementary Fig. 1d). Lastly, the Human Protein Atlas (HPA) database was used to detect the expression of CD47 in human normal tissues. Representative IHC images of CD47 expression in BRCA, HNSC, LUSC, OV, SKCM. (Supplementary Fig. 1e). From the protein expression level, it was again proved that CD47 was highly expressed in tumor tissues, especially on the cell membranes of tumor cells.

### CD47 is related to tumor immune evasion through infiltration by T lymphocyte cells

**Fig. 3:**
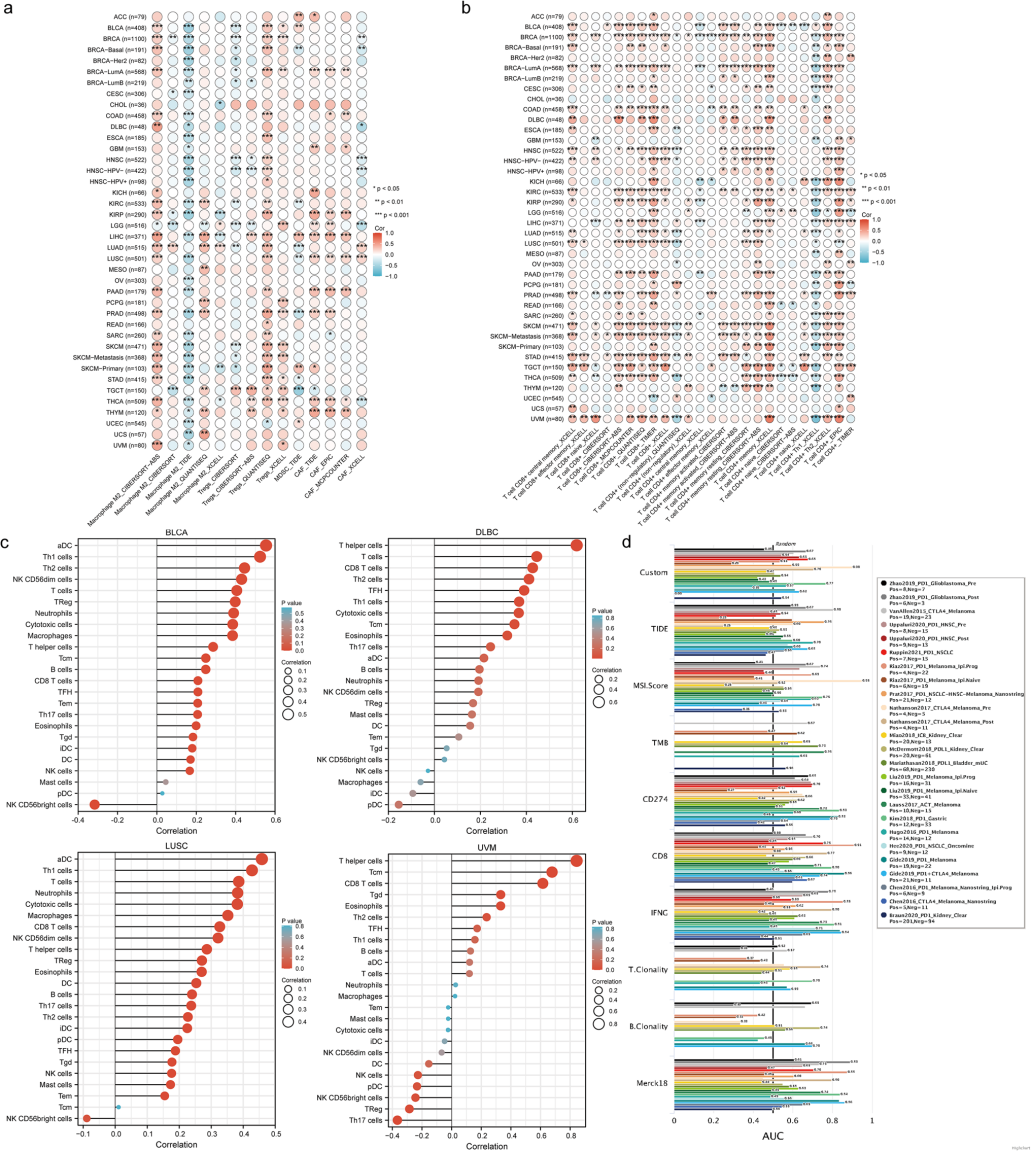
The differential expression of CD47 in tumor microenvironment. **A**, **B** The heatmap chart showed correlations of CD47 expression with infiltration by different immune cell types and different immunosuppressive cell types in various TCGA cancer types. **C** The differential expression of CD47 in Pan-cancer was predominantly associated with CD8 + T cells, CD4 + T cells, DC cells, and macrophages, as demonstrated by the lollipop. Correlation is depicted with a purity-corrected partial (**D**) Bar plot showing the biomarker relevance of CD47 compared to standardized cancer immune evasion biomarkers in immune checkpoint blockade (ICB) sub-cohorts. The AUC was utilized to assess the predictive efficacy of the test biomarkers in determining the ICB response status. Spearman’s rho values and statistical significance were used. (A. B TIMER database) (C. R3.6.3 ssGSEA). Significance representation: ns, p ≥ 0.05; *, p < 0.05; **, p < 0.01; ***, p < 0.001

Due to its association with tumor immunity evasion, we assessed the associations between CD47 expression levels and the infiltration of MDSCs, CAFs, M2-TAMs, and Treg cells through six algorithms (QUANTISEQ, XCELL, CIBERSORT, CIBERSORT-ABS, TIDE, MCPCOUNTER). These types of immune cells could promote T-cell exclusion. Treg and CAF in BRCA-LumA, Treg and CAF in LICH, Treg, MDSC, and CAF in PRAD, and Treg and CAF in THYM were found to positively correlate with CD47 expression. (r > 0.2, p < 0.05, every cell type ≥ 2 algorithms positive, and at least two types of these cells are positive) (Fig. 3a).

Subsequently, we employ the identical cognitive approach to identify the association with T cell infiltration and expression of CD47 through seven different algorithms (QUANTISEQ, XCELL, CIBERSORT, CIBERSORT-ABS, EPIC, MCPCOUNTER, TIMER). The results showed that CD47 expression was positively correlated with infiltration of CD8 + T cell in almost all cancer types. (BLCA, BRCA, BRCA-Basal, COAD, DLBC, ESCA, KIRC, KIRP, LIHC, LUSC, PAAD, PRAD, READ, SKCM, SKCM-Metastasis, STAD, TGCT, THCA, UVM) (r > 0.2, p < 0.05, at least two types of these cells are positive, and at least two types of calculation methods). Interestingly, the infiltration of CD8 + T cell effector memory is comparatively lower in the majority of cancer species compared to other types such as CD8 + T cell central memory and CD8 + T cell naive. As for CD4 + T cell, there were still a lot of CD4 + Tcells closely related to CD47, but it depends on the types of CD4 + T cell. The presence of CD47 in the majority of cancer types was observed to have a positive correlation with the infiltration of CD4 + T cell memory resting and CD4 + T cell Th2, in contrast to CD4 + T cell (non-regulatory) and CD4 + T cell Th1(Fig. 3b).

We further explored which types of T-cell infiltration in tumors were most associated with CD47 using another way. It is worth mentioning that the correlation between CD8 + T cells infiltration and CD47 is the highest in these cancer types (COAD, DLBC, ESCA, HNSC-HPV-, LIHC, LUAD, LUSC, PAAD, STAD) (Fig. 3b). CD47 expression had strong positive correlations with T cell infiltration in various cancer types including BLCA, CHOL, COADREAD, DLBC, GBM, HNSC, KIRC, KIRP, LAML, LUSC, PAAD, PRAD, SKCM, TGCT, READ, STAD, UCS, and UVM. The T cell subtypes that displayed significant associations included T helper cells, CD8 + T cells, CD4 T cells, Cytotoxic cells, Th1, Th2, Th17, as well as Tgd, Tcm, Tem, and TFH. (Fig. 3c, Supplementary Fig. 2a). Furthermore, it suggested a strong association between the infiltration of CD8 + T cells and CD47 in numerous cancer types (DLBC, ESCA, LUSC, OV, SKCM, STAD, TGCT, THCA, UCS, UVM). (r > 0.2, p < 0.05).

Then, we assessed CD47 biomarker relevance by comparing CD47 with standardized biomarkers based on its response outcomes to ICB sub-cohorts and OS predictive ability. Interestingly, we found that in 16 of the 25 ICB sub-cohorts, CD47 alone had an area greater than 0.5% under the AUC. CD47 was predicted to be more valuable than TMB, T. Clonality, B. Clonality, and MSI. Seven, nine, seven, and 13 ICB subgroups had more significant AUC values than 0.5. However, CD47 is lower than CD274, TIDE, IFNG, CD8, and Merck18. Based on these results, it is strongly indicated that CD47 plays a pivotal role in the immune microenvironment of tumors and exhibits a strong association with T-cell infiltration(Fig. 3d). Lastly, We analysed the correlation of CD47 in pan-cancer with other immune checkpoints including immunoinhibitor molecule, MHCmolecule and immunostimula molecule. We found CD47 had strong correlation with CD274, LAG3, IDO1 and TNF receptor superfamily in pan-cancer(r > 0.2, p < 0.05)( Supplementary Fig. 2b).

### CD47 on CD8 + T cells infiltration had an impact on tumor prognosis.

**Fig. 4:**
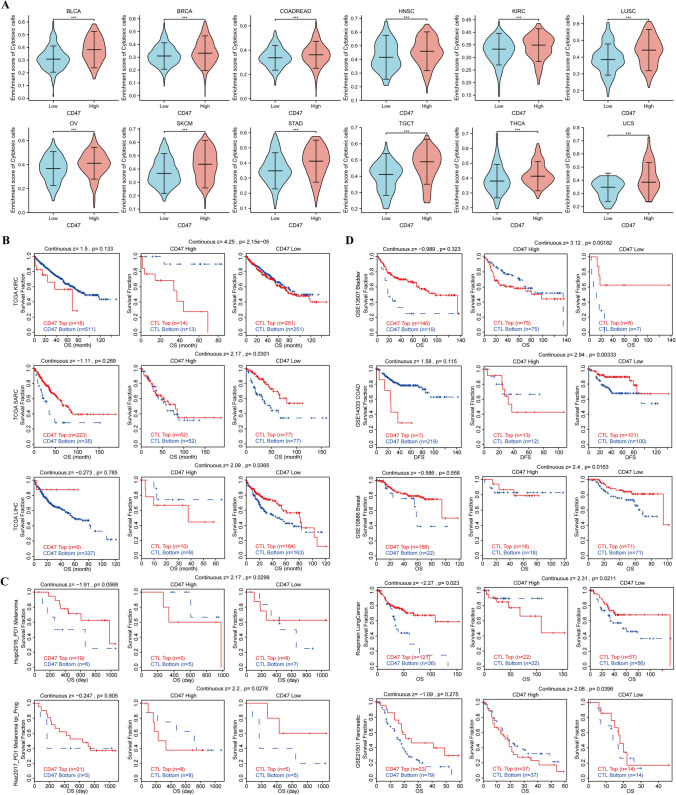
CD47 on CD8 + T cells Infiltration Influenced Tumor Prognosis. **A** The violin diagram illustrates the disparity in CD47 expression within the infiltration of Cytotoxic CD8 + T-cell. Kaplan–Meier curves (the first picture on the left) of survival ratios as a measure of the **B**TCGA cohorts **C** immunotherapeutic response (immune checkpoint blockade) between cancer cohorts **D** GEO and other cohorts with high and those with low expression levels of CD47. The remainder (the second and third picture on the left) of the graph illustrates the prognosis for the two different sets of CTL expressions with CD47-high group and CD47-low group in above cohorts. Only cancers with statistically significant differences between the cohorts are presented. Significance representation: ns, p ≥ 0.05; *, p < 0.05; **, p < 0.01; ***, p < 0.001

Drawing from our prior findings, it can be inferred that the presence of CD8 + T cells exhibited a strong correlation with CD47. Interestingly, we found that in the CD47 High group, the level of Cytotoxic CD8 + T cell was more frequently observed in BLCA, BRCA, CESC, COAD, COADREAD, ESAD, ESCA, GBM, HNSC, KIRC, LUSC, OV, SKCM, STAD, TGCT, THCA, UCS types compared to the CD47 low group based on TCGA database. (Fig. 4a, Supplemental Fig. 3a) Then we further identified the same conclusion in Multiple immunotherapy cohorts (Nathanson2017_CTLA4-OS, Gide2019_PD1-OS, Gide2019_PD1 + CTLA4-OS, Miao2018_ICB-OS, Mariathasan2018_PDL1-OS, Riaz2017_PD1-OS, Liu2019_PD1-OS Zhao2019_PD1-OS, VanAllen2015_CTLA4-OS) (Supplemental Fig. 3b).

Furthermore, it was observed that the Cytotoxic CD8 + T cell Top group had a poorer prognosis in CD47-high group than the CD47-low group (Fig. 4b). Especially in these cohorts, high Cytotoxic CD8 + T cell infiltration did not suggest a better prognosis. In the same, this phenomenon was also shown in GSE13507@PRECOG Bladder, GSE10886@PRECOG Breast, Roepman Lung Cancer @PRECOG cohorts, GSE17536 Colorectal OS, E-MTAB-3267-Kidney, GSE31684-Bladder, OV GSE31245@PRECOG, GSE49997 OV, METABRIC BreastLumA, Prostate GSE16560@PRECOG, Gide2019-PD1 + CTLA4 Melanomas (Fig. 4c, Supplemental Fig. 3c). The same results happened in KIRC, SARC, LIHC in TCGA database (Fig. 4d). It is well known that T cell dysfunction can negatively impact the prognosis even in the presence of cytotoxic CD8 + T cell, while T cell rejection might negatively impact the prognosis because of the absence of cytotoxic CD8 + T cell infiltration. Consequently, we strongly suggest that CD47 might impair CD8 + T cell function and so negatively impact tumor patients' prognosis.

### CD47 differential genes enrichment in CD8 + Tex pathway.

**Fig. 5:**
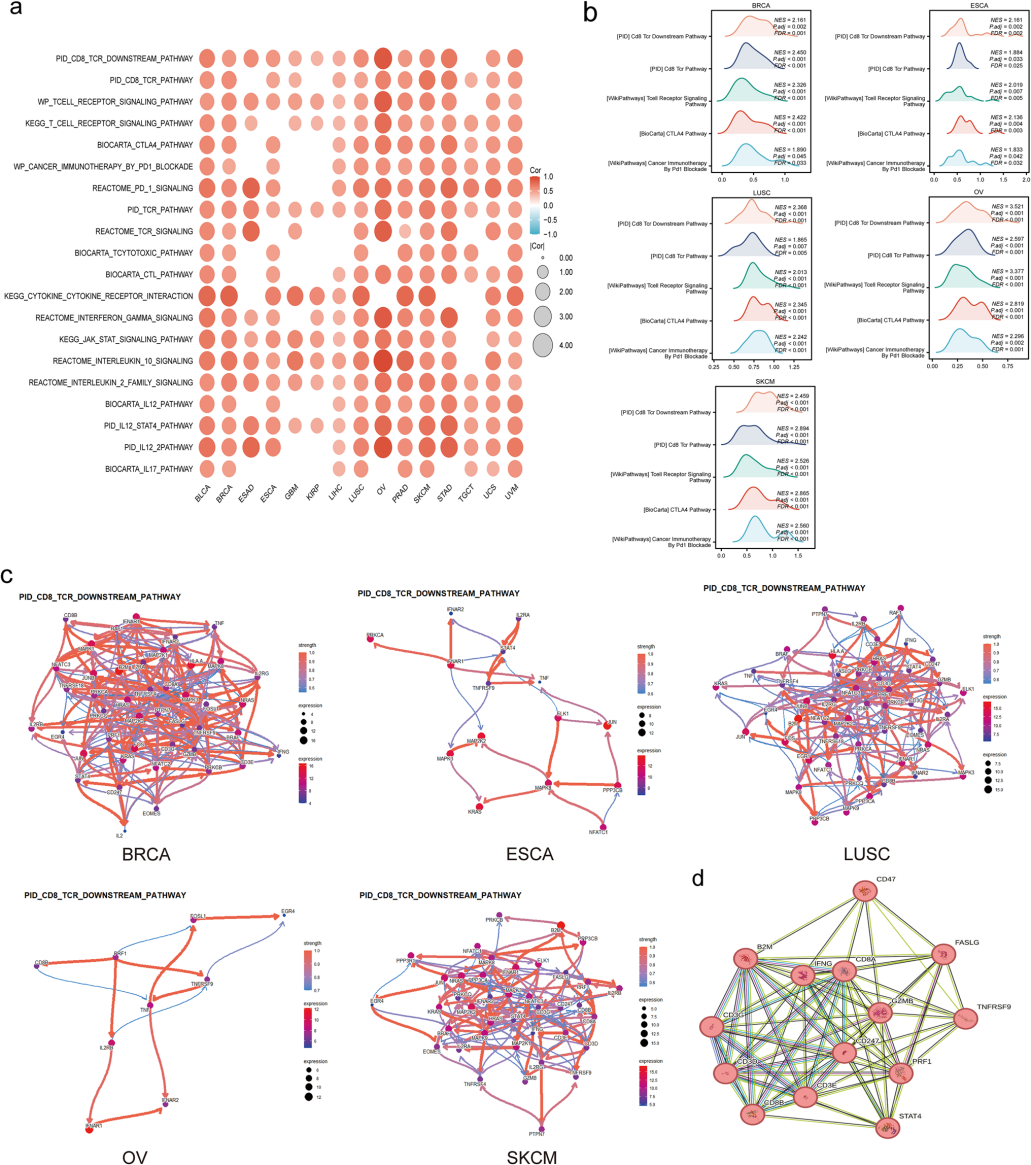
CD47 Differential Genes Enriched in CD8 + Tex Pathway. (**A**) GSEA enrichment analysis results based on CD47 differentially expressed genes in Pan-cancer. (**B**) The mountain map showcased the path enrichment outcomes, encompassing PID CD8 TCR Downstream Pathway, PID CD8 TCR Pathway, WP Tcell Receptor Signal Pathway, BIOCARTA CTLA4 Pathway, and WP Cancer Immunotherapy by PD1 Blockade, along with the gene regulatory network for the PID CD8 TCR Downstream Pathway. (**D**) The PPI network demonstrated the correlation between the CD47 protein and the central protein of PID CD8 TCR Downstream Pathway. The representation of significance is as follows: ns, p ≥ 0.05; *, p < 0.05; **, p < 0.01; ***, p < 0.001

We categorized the TCGA cohort data into high-expression group and low-expression group, and performed pathway enrichment analysis for both groups of differently expressed genes (p < 0.05). We found that some of the same pathways are present in cancers(Fig. 5a), which includes PID CD8 TCR Downstream Pathway in BLCA, BRCA, ESAD, ESCA, GBM, KIRP, LIHC, LUSC, OV, PRAD, SKCM, STAD, UCS, UVM; PID CD8 TCR Pathway in BLCA, BRCA, ESCA, GBM, LIHC, LUSC, OV, PRAD, SKCM, STAD, TGCT, UCS, UVM; WP T cell Antigen Receptor TCR Signal Pathway in BLCA, BRCA, ESCA, GBM, KIRP, LIHC, LUSC, OV, PRAD, SKCM, STAD, TGCT, UCS, UVM; BIOCARTA CTLA4 Pathway and WP Cancer Immunotherapy By PD1 Blockade in BLCA, BRCA, ESCA, LIHC, LUSC, OV, PRAD, SKCM, STAD, TGCT, UCS, UVM. Furthermore, CD8 + Tcell exhaustion correlated pathways comprise IL2, IL10, IL12, IL17, INF-gamma, T cell, TCR, and JAK-STAT.

Furthermore, mountain maps presented a visualization of the above first 5 pathways enrichment results (Fig. 5b), in BRCA, ESCA, LUSC, OV, and SKCM to further demonstrate the distribution of corresponding numbers of differential genes enriched. The figure illustrates that NES (normalized enrichment score) exhibited positivity, with the majority of the differential genes exhibiting enrichment in the high-expression group. We explore further the gene regulatory network of PID CD8 TCR Downstream Pathway (Fig. 5c, Supplemental Fig. 4a), we discovered that TNFRSF9 and CD8A were expressed at a high level in above all cancers. Taking into account the regulatory networks deduced from the enriched outcomes, we can direct our attention towards a regulatory pathway extending from TNFRSF9 to IL2RA/B/G, via CD8A. Subsequently, we employ PPI(Fig. 5d) to determine the association between the CD47 protein and the proteins linked to the core-enriched molecules. The findings indicated a direct interaction between CD47 protein and CD8A, TNFRSF9, IFNG, B2M, and GZMB. In light of our observations, we deduced that the activation of the CD47 within the signaling pathway could potentially exert a crucial influence on the regulation of CD8 + T cell functionality—a phenomenon that may potentially collaborate with TNFRSF9.

### CD47 expression is related to the CD8 + Tex in pan-cancer.

**Fig. 6:**
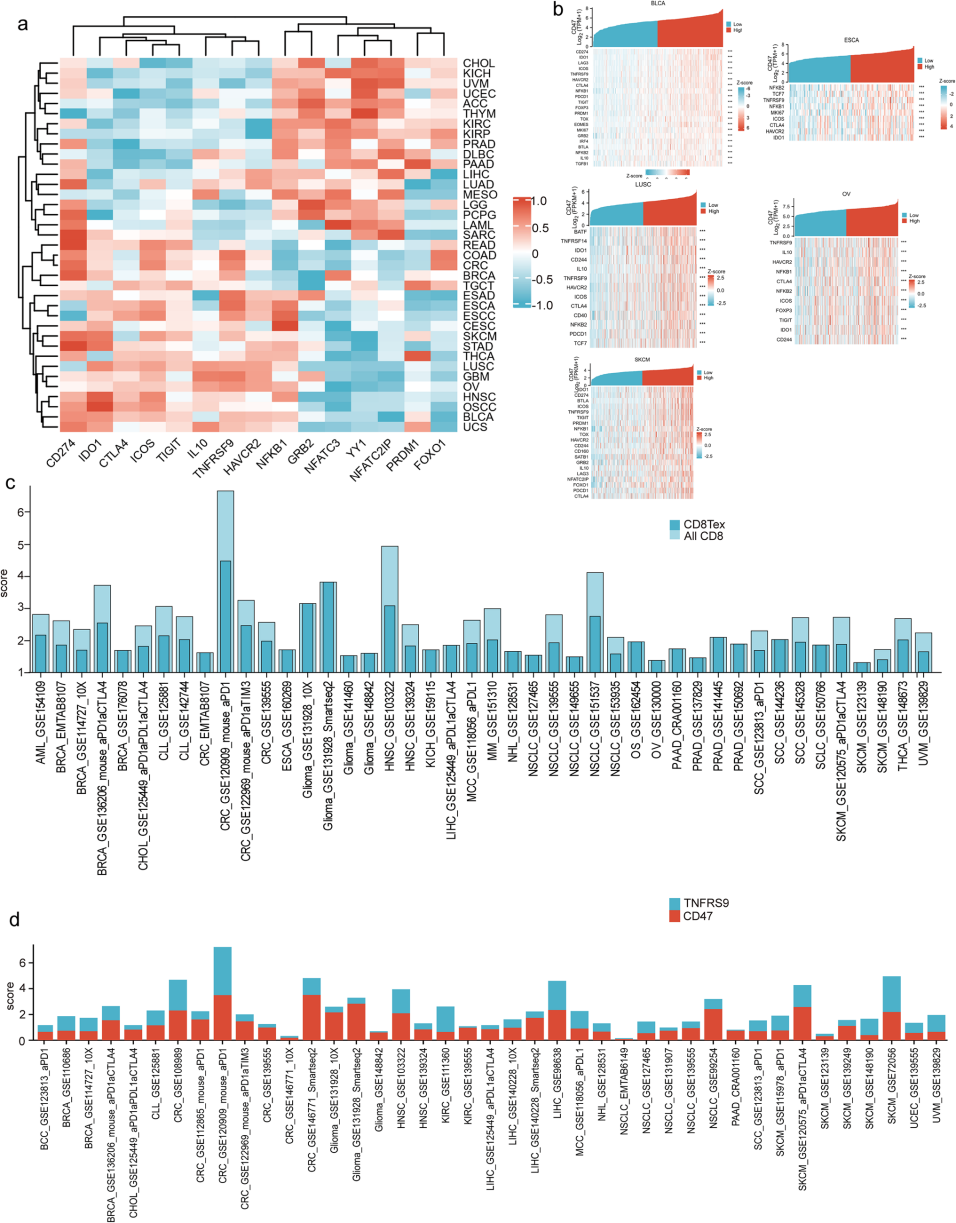
CD47 expression is related to the CD8 + Tex in many cancer types. **A**, **B** Co-expression heat map shows the relationship between the expression of CD47 in pan-cancer and the exhausted genes of T cells, especially TNFRSF9, CD274, IDO1, and ICOS. **C** The bar chart displayed a wide range of CD47 expression in CD8 + Tex and CD8 + T cell from a single-cell database in a pan-cancerous environment. (**D**) The histogram illustrates the manifestation of CD47 and TNFRSF on CD8 + Tex.Significance representation: ns, p ≥ 0.05; *, p < 0.05; **, p < 0.01; ***, p < 0.001

We delved deeper into the connection between CD47 and the exhaustion of T-cells. The co-expression heat map showed a relationship between CD47 expression in pan-cancer and T-cell exhausted genes. [45] CD274, IDO1, CTLA4, ICOS, TIGIT, IL10, TNFRSF9, HAVCR2 exhibited significant co -expression with CD47 in BLCA, BRCA, CESC, COAD, READ, CRC, ESCA, ESCC, GBM, HNSC, LUSC, OSCC, OV, SKCM, STAD, TGCT, THCA, UCS; NFKB1, GRB2, NFATC3, YY1, NFATC2IP, PRDM1, and FOXO1 in other cancers. Additionally, it was demonstrated that TNFRSF9 ranked among the top three genes in the tumors listed below: BRCA, COAD, ESCA, ESAD, ESCC, GBM, LUSC, OV, and DLBC(r > 0.5 but not the top); whereas CD274 held the top three positions in BLCA, BRCA, CESC, COAD, READ, CRC, LAML, LGG, LUAD, OSCC, PCPG, PRAD, READ, SARC, SKCM, STAD, THCA, UCS (Supplemental Table 1). (Fig. 6a, b, Supplemental 4c).

After that, we used the single-cell analysis to evaluate the expression of CD47 in CD8 + Tex and CD8 + T cells from the TISCH database and used the embedded bar chart to show the Data distribution. We found CD47 mainly expressed on CD8 + Tex(> 50%) in AML, BRCA, CHOL, CLL, CRC, ESCA, Glioma, HNSC, KICH, LIHC, MCC, MM, NHL, NSCLC, OS, OV, PRAD, SCC, SCLC, SKCM, THCA, UVM (Fig. 6c). And we further detect the expression of TNFRSF9 and CD47. It was observed that both of them exhibited expression on CD8 + Tex in BRCA, BCC, CHOL, CLL, CRC, Glioma, KIRC, LIHC, MCC, NHL, NSCLC, PAAD, SKCM, UCEC, and UVM (Fig. 6d). The findings indicate that the association of CD47 and TNFRSF9 with CD8 + Tex in pan-cancer suggests their involvement in the dysregulation of CD8 + Tex in this type of cancer.

## Discussion

These findings highlight a strong association between CD47 and the initiation and progression of multiple cancer types. Previous studies have also documented functional associations between CD47 and tumorigenesis, including its role in maintaining immune system homeostasis [2, 46]. Furthermore, our investigation focuses on examining the correlation between CD47 expression levels and various parameters such as prognosis [48, 49], the tumor microenvironment (TME) [50], immune escape mechanisms [51], adaptive immunity [47], and notably, T-cell exhaustion [52], within the pan-cancer landscape of TCGA. The substantial heterogeneity and distinctive clinical characteristics observed across different cancer types and subtypes carry significant implications for further research and clinical applications.

In this study, we investigated the oncogenic role of CD47 within TCGA dataset. Our results reveal that CD47 mRNA or protein levels exhibit notable overexpression across a broad spectrum of TCGA cancers, prominently including BRCA, CHOL, COAD, ESCA, HNSC, KIRC, LIHC, PAAD, STAD, THCA, and UCEC. Furthermore, high levels of CD47 were associated with advanced tumor staging or poorer prognosis in ACC, HNSC, KICH, KIRC, LGG, LICH, LUSC, OV, PAAD, RCC, THCA, UCEC, and UVM. These findings corroborate existing clinical and preclinical evidence highlighting elevated CD47 expression in cancer and its correlation with high-risk tumor features [45, 53–55]. Our observations suggest CD47 as a potential biomarker for cancer diagnosis, staging, and post-treatment monitoring.

Subsequently, we evaluated the relationship between the expression levels of CD47 and the infiltration of four immunosuppressive cell populations. Specifically, these cell types, including CAFs, Tregs, M2-TAMs, and MDSCs, have been recognized as biomarkers associated with T-cell exclusion within the tumor microenvironment (TME) [56–59].The interaction between CAFs and CD47 involves the receptor THBS2/THBS3 on CAFs interacting with CD47 expressed on cancer cells, promoting further cancer progression. Another receptor on the cell membrane of CAFs, MDK, interacts with NCL/SDC2/SDC on cancer cells, potentially serving as therapeutic targets [60]. Regarding the current understanding of the relationship between regulatory T cells (Tregs) and CD47, research has indicated that the ligand of CD47, SIRPγ, is expressed on T cells and varies with differentiation. Some studies have shown that although SIRPγ is expressed on Tregs, it does not participate in their suppressive function [61]. Regarding M2-TAMs, reprogramming TAMs into pro-inflammatory M1 macrophages or inhibiting the M2 polarization of macrophages can disrupt the interaction between tumor cells and macrophages, thereby influencing immune function. Furthermore, it has been reported that the CD47/SIRPα axis plays a crucial role in mediating the interaction involving MDSCs in this context [62]. Inhibition of CXCR2 in G-MDSCs augments the efficacy of CD47 blockade in promoting melanoma tumor cell clearance [63]. Interestingly, we observed a robust correlation between the expression levels of immunosuppressive cells and CD47 in BRCA-LumA, LICH, LUAD, PRAD, and THYM. (≥ 2 immunosuppressive cell types, every cell type ≥ 2 calculated method positive, r > 0.2 and p < 0.05). Therefore, we hypothesize that one of the primary mechanisms through which CD47 regulates tumor immune evasion, tumor progression, and metastasis is through T-cell rejection.

Consequently, our investigation into the association between CD47 and T-cell infiltration utilized seven distinct algorithms (QUANTISEQ, XCELL, CIBERSORT, CIBERSORT-ABS, EPIC, MCPCOUNTER, TIMER, including ≥ 2 immune types, every cell type ≥ 2 calculated method positive, r > 0.2, p < 0.05). These findings demonstrated significant positive correlations in BLCA, BRCA, BRCA-Basal, COAD, DLBC, ESCA, KIRC, KIRP, LIHC, LUSC, PAAD, PRAD, READ, SKCM, SKCM-Metastasis, STAD, TGCT, THCA, UVM. CD47 blockade has been implemented in various models and clinical trials to enhance phagocytosis, augment T cell infiltration into tumors, and reduce tumor burden both in vitro and in vivo [64, 65]. For example, the co-delivery nanocarrier aCD47-DMSN was designed by encapsulating DOX within the mesoporous cavity of MSN and adsorbing aCD47 onto the MSN surface. Following intravenous administration, aCD47-DMSN exhibited robust antitumor efficacy by enhancing the infiltration of CD8 + T cells into the tumor. These results offer another hypothesis that CD47 plays a role in activating dysfunctional T-cell phenotypes to regulate immune escape mechanisms and patient outcomes [66–68].

While the prognostic implications of CD47-mediated CD8 + T cell activity are yet to be fully elucidated, our focus was on examining the relationship between CD47 expression and levels of infiltrating cytotoxic CD8 + T cells. Analysis of the TCGA database revealed a notable increase in cytotoxic CD8 + T cell infiltration in BLCA, BRCA, CESC, COAD, COADREAD, ESAD, ESCA, GBM, HNSC, KIRC, LUSC, OV, SKCM, STAD, TGCT, THCA, and UCS when comparing the CD47 high and low expression groups. Subsequently, we investigated the potential impact of these findings on treatment response and patient outcomes. Interestingly, no significant differences in prognosis were observed based on CD47 expression status alone. Thus, we further assessed the prognostic implications of cytotoxic CD8 + T cell infiltration in both high and low CD47 expression groups. Notably, in cases where CD47 expression was high, we also explored the prognostic significance of cytotoxic CD8 + T cell infiltration in both the CD47-high and CD47-low subgroups across BLCA, BRCA, COAD, KIRC, LIHC, LUNG CANCER (Adeno, Large, Squamous), OV, PAAD, PRAD, ARC, SKCM, TCGA, PRECOG, and the METABIC cohort. It is widely recognized that the efficacy of immunotherapy is closely associated with the infiltration of cytotoxic CD8 + T cells [66–68] (Supplemental Fig. 3a), and good Cytotoxic CD8 + T cell infiltration usually represents a better prognosis [69]. CD47 has been shown to impede the recruitment and activation of effector T cells, leading to intratumoral immunosuppressive effects. By blocking CD47, the suppression of cytotoxic CD8 + T cell function is alleviated, thereby sustaining the anti-tumor response. This blockade operates indirectly by thwarting immunosuppressive signals expressed in antigen-presenting cells or by shielding tumor-infiltrating cytotoxic CD8 + T cells from the local tumor microenvironment's irradiation [20]. Hence, the expression level of CD47 was associated with dysfunctional T-cells in those cohorts.

Then, utilizing TCGA data, we stratified these tumors into high and low-CD47 expression groups and identified differentially expressed genes between the two cohorts based on median CD47 expression levels. These genes were subjected to pathway enrichment analysis to uncover the correlation between high CD47 expression and immunomodulatory effects. High CD47 expression in BLCA, BRCA, ESAD, ESCA, GBM, KIRP, LIHC, LUSC, OV, PRAD, SKCM, STAD, UCS, and UVM was associated with pathways involving interactions among CD8 + T cells, cytokines, and key immunomodulators such as PD-1, CTLA4, IL-10, INF gamma, and cytotoxic CD8 + T cells. These findings suggest that CD47 may play a crucial role in modulating adaptive immunity, particularly in the context of CD8 + T cell exhaustion. Notably, PD-1, a well-established checkpoint pathway present in both immune and tumor cells, was among the pathways implicated in this immunomodulatory network [70]. The blockade of PD-1 signaling influences the TME [71] by initiating an immune response within tumor cells. Pathways induced by PD-1 blockade in cancer immunotherapy have been closely linked to the upregulation of CD47 expression, indicating that elevated CD47 levels post PD-1 inhibitor treatment lead to a reduction in CD8 + T cells, ultimately contributing to drug resistance. Targeting these factors represents a promising adjunctive strategy for patients undergoing anti-programmed death-1 receptor (PD-1)/anti-programmed death-ligand 1 (PD-L1) therapy. Additionally, IL-10 has been demonstrated to contribute to T cell dysfunction. CD8 + T cells play a pivotal role in immune response, metastasis, and tumorigenesis [72]. A notable association was observed between IL-2 levels and the prognosis of patients diagnosed with pan-cancer [73, 74]. However, our analysis did not reveal a significant correlation with T-dysfunction-related pathways in other cancer types. This discrepancy could potentially be attributed to limited data availability or other factors, such as the absence of a key regulatory gene like LAYN that modulates T cell function, or variations in the TME [75].

To enhance comprehension of the regulatory network, we focused on the enriched pathway-PID CD8 TCR DOWNSTREAM PATHWAY. Utilizing Bayesian network inference through CBNplot, we identified heightened expression of TNFRSF9 and established a regulatory pathway connecting CD8A, IL2RA/B/G to TNFRSF9 in BRCA, ESCA, LUSC, OV, and SKCM. This led to the inference that CD47 might modulate the CD8 TCR DOWNSTREAM pathway, inducing CD8 + T cell exhaustion, potentially in conjunction with TNFRSF9. Subsequently, employing protein–protein interaction (PPI) analysis (Fig. 5d), we elucidated a direct interaction between the CD47 protein and the proteins associated with the core-enriched molecules. Furthermore, investigation into the CD47 ligand SIRPA and the PPI network of the pathway core genes revealed its association with IL2, IL2RA, CD8A, B2M, IFNG, and GZMB, thereby substantiating our deduction. (Supplemental Fig. 4b) (all combined score > 0.3).

Additionally, we conducted an analysis of the expression profile and co-expression patterns of the T-cell depletion gene set [43] CD47 across pan-cancer cohorts. Notably, CD274, IDO1, CTLA4, ICOS, TIGIT, IL10, TNFRSF9, and HAVCR2 demonstrated significant co-expression with CD47 in Group 1 (BLCA, BRCA, CESC, COAD, READ, CRC, ESCA, ESCC, GBM, HNSC, LUSC, OSCC, OV, SKCM, STAD, TGCT, THCA, UCS), while NFKB1, GRB2, NFATC3, YY1, NFATC2IP, PRDM1, and FOXO1 exhibited co-expression in other cancer types designated as Group 2. Noteworthy findings included TNFRSF9 ranking among the top three co-expressed genes in BRCA, COAD, ESCA, ESAD, ESCC, GBM, LUSC, OV, and DLBC (with correlation coefficients exceeding 0.5 but not ranking first), and CD274 ranking among the top three in BLCA, BRCA, CESC, COAD, READ, CRC, LAML, LGG, LUAD, OSCC, PCPG, PRAD, READ, SARC, SKCM, STAD, and THCA. These observations suggest potential functional partnerships between these genes and CD47 across various cancer types, a phenomenon also corroborated by previous studies in select cancer types [24, 76, 77].

The TME is widely known for its substantial heterogeneity [78]. Utilizing the TISCH single-cell database, we explored the impact of CD47 on the TME. Notable variations in immune cell profiles were observed across primary and metastatic tumor sites. Specifically, our analysis revealed that CD47 was prominently expressed on CD8 + exhausted T cells (CD8 + Tex), exhibiting significantly higher expression levels compared to other CD8 + T cell subsets in AML, BRCA, CHOL, CLL, CRC, ESCA, Glioma, HNSC, KICH, LIHC, MCC, MM, NHL, NSCLC, OS, OV, PRAD, SCC, SCLC, SKCM, THCA, and UVM (Fig. 6c). Furthermore, in BRCA, CHOL, CLL, CRC (COADREAD), GBM, HNSC, KIRC, LIHC, MCC, NHL, NSCLC, PAAD, SCC, SKCM, UCEC, and UVM, both CD47 and TNFRSF9 were co-expressed on CD8 + exhausted T cells. These findings suggest a potential functional partnership between CD47 and TNFRSF9 in regulating CD8 + exhausted T cells, particularly in BRCA, ESCA, LUSC, OV, and SKCM (Supplemental Table 2).

Currently, extensive research efforts are focused on the development of dual-targeting strategies involving CD47, with notable progress in the development of CD47 and TNFRSF9 targeting fusion protein (DSP107) [24, 79], PD1 and CD47 bispecific fusion molecules, bispecific antibody CD47xPD-L1, CD47 x HER2, CD47xICAM-1, and other approaches [80–84]. These novel strategies not only aim to mitigate the hematologic toxicity and adverse effects associated with CD47 monoclonal antibodies but also seek to enhance the anti-tumor efficacy of CD47 blockade. By elucidating the cell-intrinsic mechanisms regulated by CD47, we gain insights into its role in promoting tumor biology. Collectively, these advancements position CD47 as a promising therapeutic target in the treatment of both solid and non-solid tumors.

## Limitation

In our study, we encountered negative and opposite findings in certain types of cancers. The majority of the studies utilized public databases available on the internet, which may have contained slightly outdated data. Future research should focus on utilizing real-world cohorts and conducting multicenter data analysis to enhance the robustness and relevance of the findings. Additionally, the small sample size and dearth of reliable data contributed to limitations in the generalizability of the results. Moreover, methodological deficiencies, such as constraints in the technology and instruments used for data collection, further weakened the study's overall findings. The high expression levels of CD47 and TNFRSF9 in cancer cells and their correlation can be demonstrated through multiple immunofluorescence staining. Additionally, utilizing WB-PCR, it can be shown that CD47 wild-type cancer cells express a higher amount of T cell depletion factors compared to CD47 knockout cancer cells. Through T cell co-culture experiments, it can be established that CD47 knockout cancer cells exhibit reduced T cell depletion compared to wild-type cancer cells. The analysis revealed a poor prognosis in the low CD47 expression group in SKCM. Moreover, the expression of CD47 in adjacent tissues was observed to be higher than in cancer tissues, as evidenced in KICH and LUSC. These findings suggest potential factors contributing to these observations, including intrinsic tumor heterogeneity, the tumor immune microenvironment, specifically telomere Tex cell richness [85, 86], or data insufficiency, warranting further experimental validation and investigation.

## Conclusions

CD47 plays a pivotal role in the TME, prognosis, and immunotherapy not only through its interactions with macrophages but also with tumor-infiltrating T-lymphocyte cells, particularly CD8 + T cells across various cancer types. The interaction between CD47 and TNFRSF9 triggers exhaustion in CD8 + T cells, leading to an unfavorable prognosis. A therapeutic approach targeting both CD47 and TNFRSF9 has the potential to activate both innate and adaptive immune responses, presenting a significant advancement in treatment modalities, especially for patients with BRCA, ESCA, LUSC, OV, and SKCM.

### Supplementary Information

Additional file1 (ZIP 95 KB)

Additional file2 (TIF 10061 KB)

Additional file3 (TIF 131045 KB)

Additional file4 (TIF 3808 KB)

Additional file5 (TIF 4244 KB)

Additional file6 (TIF 27286 KB)

Additional file7 (TIF 13340 KB)

Additional file8 (TIF 14377 KB)

## Data Availability

The datasets provided in this study can be found in online repositories.

## Abbreviations

4-1BB: TNFRSF9 (tumor necrosis factor receptor superfamily member 9), CD137, ILA
ACC: Adrenocortical carcinoma
ALL: Acute lymphoblastic leukemia
AUC: Area under curve
BCC: Basal cell carcinoma
BIOCARTA: A subset of cancer pathway( icluding 292 gene sets)https://maayanlab.cloud/Harmonizome/dataset/Biocarta
BLCA: Bladder urothelial carcinoma
BRCA: Breast invasive carcinoma
BRCA-Lum A: LumA positive pathological type of breast invasive carcinoma
BRCA_SRP114962: SRP114962 cohort of non-small cell lung cancer
CAF: Cancer associated fibroblasts
CBNplot: Ayesian network plots for enrichment analysis
CD274: PD-L1 (Programmed death-ligand 1)
CESC: Cervical squamous cell carcinoma and endocervical adenocarcinoma
CHOL: Cholangiocarcinoma
CLL: Chronic lymphocytic leukemia
COAD: Colon adenocarcinoma
CP: Cancer pathway
CRC: Colerectal cancer
CTL: Cytotoxic T lymphocytes
CTLA4: Cytotoxic T-lymphocyte-associated protein 4
DEG: Differential Expression Analysis
DLBC: Lymphoid neoplasm diffuse large B-cell lymphoma
DSP107: A Novel Bi-Functional Fusion Protein That Combines Inhibition of CD47 with Targeted Activation of 4-1BB
DSS: Disease-Specific Survival
ESAD: Esophageal adenocarcinoma
ESCA: Esophageal carcinoma
ESCC: Esophageal cell squamous carcinoma
FCGR3A: Fc gamma receptor IIIa
FDR: False discovery rate
FPKM: Fragments Per Kilobase of exon model per Million mapped fragments
GBM: Glioblastoma multiforme
GEO: Gene Expression Omnibus (https://www.ncbi.nlm.nih.gov/geo/)
GRB2: Growth factor receptor bound protein 2
GSE: GEO Series
GTEx: Genotype-Tissue Expression Project (https://commonfund.nih.gov/GTEx)
HAVCR2: Hepatitis A virus cellular receptor 2
HER2: Human epidermal growth factor receptor 2
HTSeq: High-throughput sequence analysis (https://pypi.org/project/HTSeq/)
ICAM-1: Intercellular Cell Adhesion Molecule-1
ICB: Immune checkpoint blockade
ICOS: CD278 (Inducible Co-Stimulator)
IDO1: Indoleamine 2,3-dioxygenase 1
IFNG: Interferon γ
IL: Interleukin
JAK: Janus kinase
KEGG: Kyoto Encyclopedia of Genes and Genomes (https://www.genome.jp/kegg/)
KICH: Kidney chromophobe
KIRC: Kidney renal clear cell carcinoma
KIRP: Kidney renal papillary cell carcinoma
LAML: Acute myeloid leukemia
LAYN: Layilin
LGG: Brain lower grade glioma
LIHC: Liver hepatocellular carcinoma
LUAD: Lung adenocarcinoma
LUSC: Lung squamous cell carcinoma
M2-TAM: M2-tumor-associated macrophages
McAb: Monoclonal antibody
MCC: Merkel cell carcinoma
MDSC: Myeloid-derived suppressor cells
MESO: Mesothelioma
METABRIC: Molecular Taxonomy of Breast Cancer International Consortium (https://www.mercuriolab.umassmed.edu/metabric)
MHC: Main histocompatibility complex
MSI: Microsatellite instability
MSigDB: Molecular Signatures Database (https://www.gsea-msigdb.org/gsea/msigdb/)
MYC: Gene
NFATC2IP: Nuclear factor of activated T cells 2 interacting protein
NFATC3: Nuclear factor of activated T cells 3
NF-κB: Nuclear factor κB
NHL: Non-Hodgkin lymphoma
NS: No significance
NSCLC: Non-small cell lung cancer
NSCLC_EMTAB6149: EMTAB6149 cohort of non-small cell lung cancer
OS: Overall survival
OSCC: Oral squamous cell carcinoma
OV: Ovarian serous cystadenocarcinoma
PAAD: Pancreatic adenocarcinoma
PCPG: Pheochromocytoma and paraganglioma
PD1: Programmed cell death protein 1
PFI: Progress Free Interval
PID: A subset of cancer pathway( icluding 196 gene sets)
PPI: Protein–Protein Interaction Networks
PRAD: Prostate adenocarcinoma
PRDM1: PR domain zinc finger protein 1
PRECOG: PREsentation and Characterization Of Growth-data (https://precog.stanford.edu/)
PTEN: Phosphatase and tensin homolog
REACTOME: A subset of cancer pathway (browse 1615 gene sets) https://reactome.org/
READ: Pectum adenocarcinoma
SARC: Sarcoma
SCC: Squamous cell carcinoma
SEEA: System of Environmental Economic Accounting
SIRPα: Signal regulatory protein α
SKCM: Skin cutaneous melanoma
STAD: Stomach adenocarcinoma
STAT: Signal transducer and activator of transcription
STRING: A database of functional protein association networks
TCGA: The Cancer Genome Atlas
Tcm: Central memory T cell
TCR: T cell receptor
Tem: Effector memory T cell
Tex: Exhausted T cell
Tfh: Follicular helper T cell
TGCT: Testicular germ cell tumors
Tgd: Gamma delta T cell
Th: T helper cells
THCA: Thyroid carcinoma
THYM: Thymoma
TIDE: Tumor immune dysfunction and exclusion
TIM: Translocation induced circling mutation
TIME: Tumor immune microenvironment
TIMER: Tumor immune estimation resource
TISCH: Tumor Immune Single-cell Hub
TLR: Toll-like receptors
TMB: Tumor mutational burden
TME: Tumor microenvironment
TNF: Tumor necrosis factor
TPM: Transcripts per million reads
Treg: Regulatory T cell
TTI-621: SIRPα Fc
UCEC: Uterine corpus endometrial carcinoma
UCS: Uterine carcinosarcoma
UVM: Uveal melanoma
WT: Wild type
WP: A subset of cancer pathway (icluding 664 gene sets)
XENA-TCGA GTEx: Xena.ucsc.edu
YY1: YIN-YANG-1
