# Supplementary material for: Pan-cancer analysis for the prognostic and immunological role of CD47: interact with TNFRSF9 inducing CD8 + T cell exhaustion
Source: Discov Oncol. 2024 May 8;15:149. doi: 10.1007/s12672-024-00951-z (PMC11078914; doi:10.1007/s12672-024-00951-z)
Supplement: Supplementary file 1 — Additional file1 (ZIP 95 KB) [file 12672_2024_951_MOESM1_ESM.zip › Suplemental information/Online Resource.docx]

链接：https://pan.baidu.com/s/1BC0v8D1oubeMXFX1Rmk66A

提取码：rpa4

--来自百度网盘超级会员V7的分享

Link: https://pan.baidu.com/s/1BC0v8D1oubeMXFX1Rmk66A

Extraction code: rpa4

-- From Baidu web disk super member V7 share
