# Supplementary figures and images for: Pan-cancer analysis for the prognostic and immunological role of CD47: interact with TNFRSF9 inducing CD8 + T cell exhaustion
Source: Discov Oncol. 2024 May 8;15:149. doi: 10.1007/s12672-024-00951-z (PMC11078914; doi:10.1007/s12672-024-00951-z)

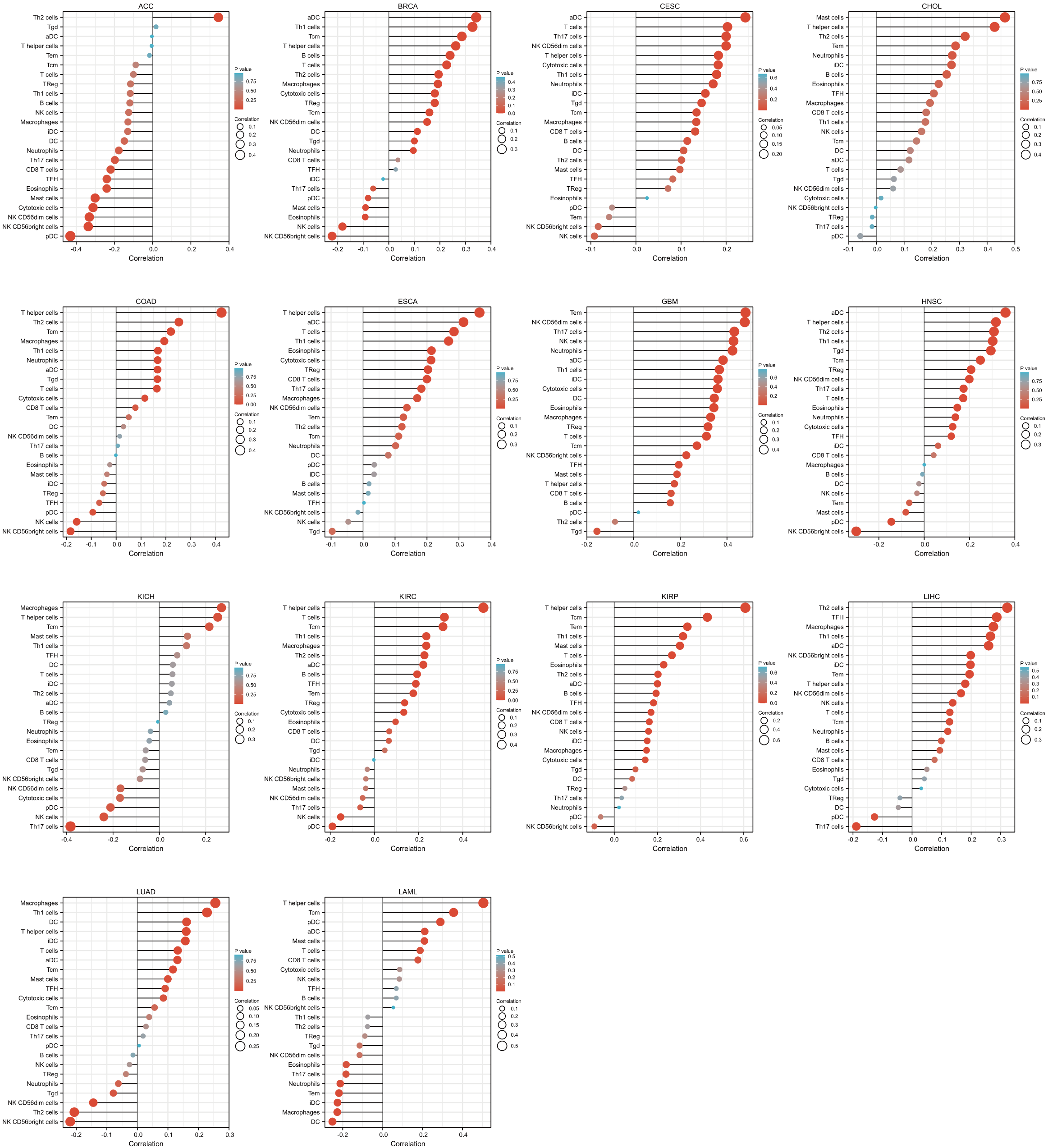

Supplement: Supplementary file 4 — Additional file4 (TIF 3808 KB) [file 12672_2024_951_MOESM4_ESM.tif]

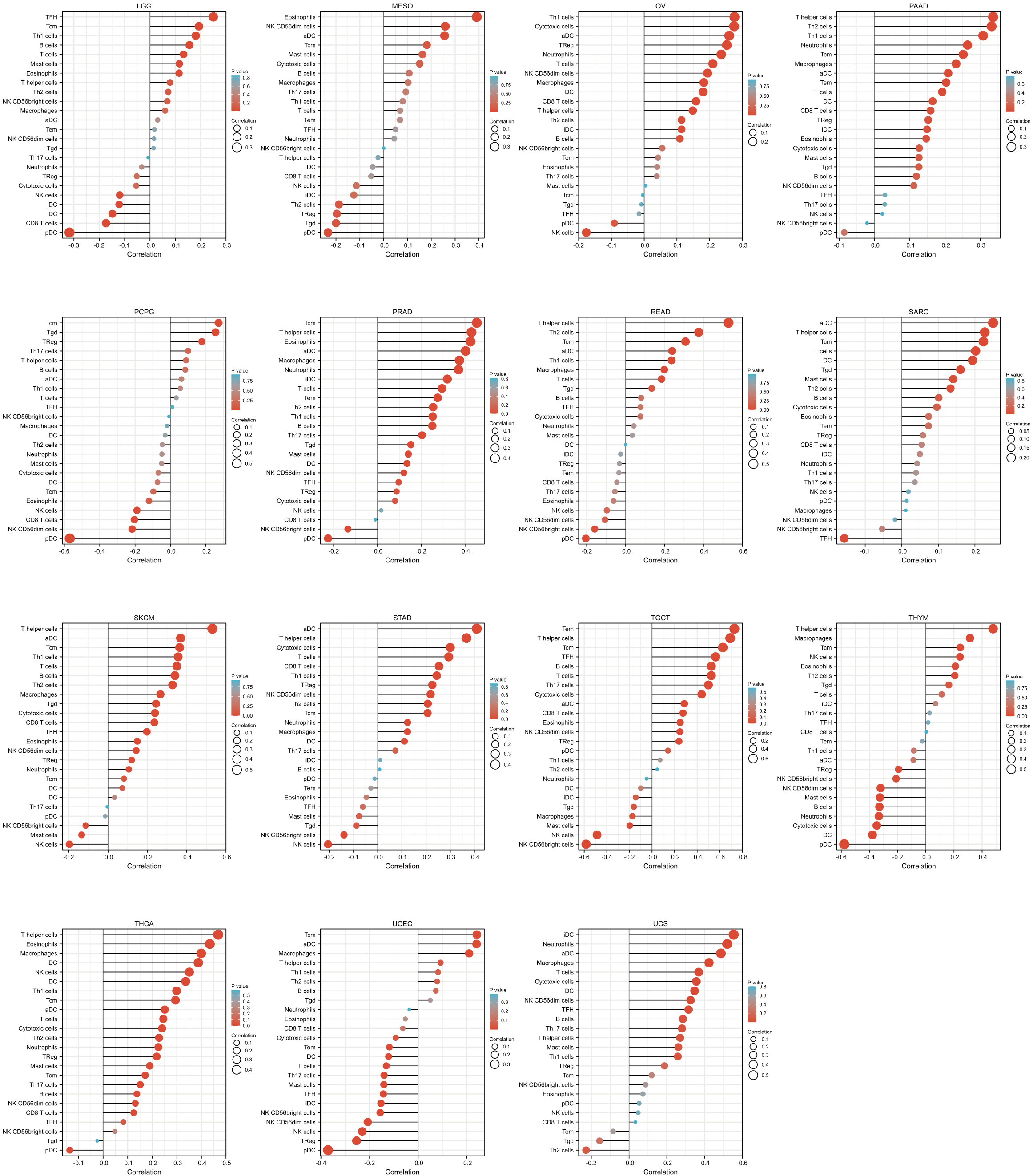

Supplement: Supplementary file 5 — Additional file5 (TIF 4244 KB) [file 12672_2024_951_MOESM5_ESM.tif]

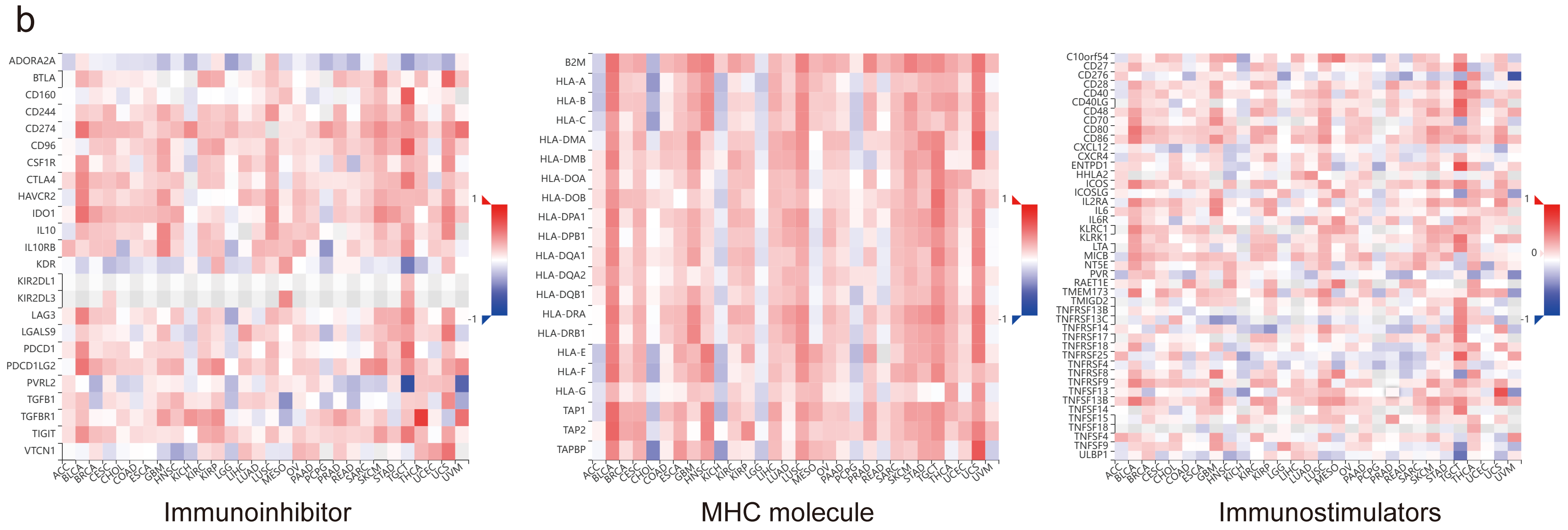

Supplement: Supplementary file 6 — Additional file6 (TIF 27286 KB) [file 12672_2024_951_MOESM6_ESM.tif]

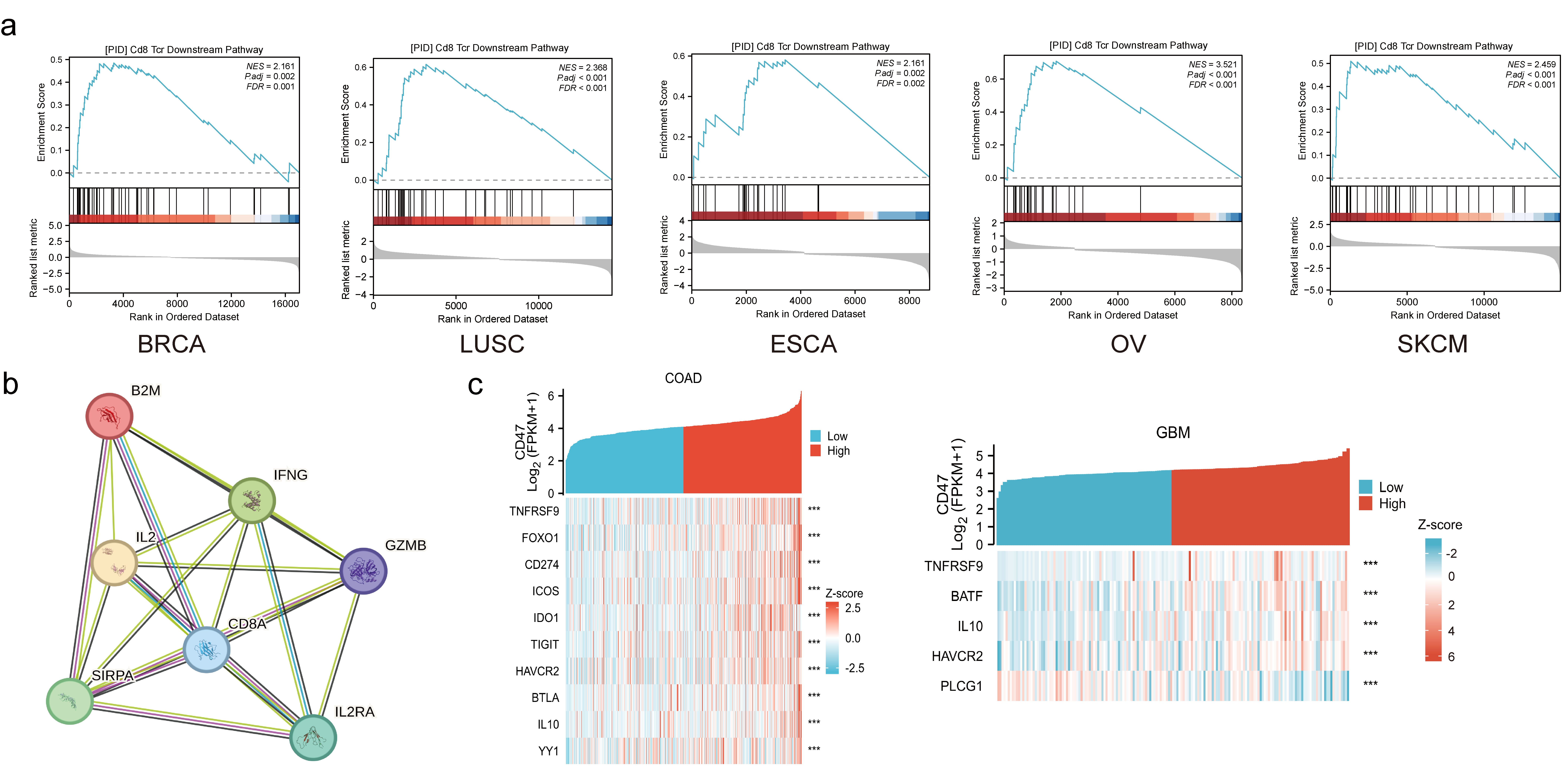

Supplement: Supplementary file 8 — Additional file8 (TIF 14377 KB) [file 12672_2024_951_MOESM8_ESM.tif]
